# Melatonin Mitigates Oxazolone-Induced Colitis in Microbiota-Dependent Manner

**DOI:** 10.3389/fimmu.2021.783806

**Published:** 2022-01-18

**Authors:** Zi-xiao Zhao, Xi Yuan, Yan-yan Cui, Jun Liu, Jing Shen, Bi-ying Jin, Bing-cheng Feng, Yun-jiao Zhai, Meng-qi Zheng, Guan-jun Kou, Ru-chen Zhou, Li-xiang Li, Xiu-li Zuo, Shi-yang Li, Yan-qing Li

**Affiliations:** ^1^ Department of Gastroenterology, Qilu Hospital, Cheeloo College of Medicine, Shandong University, Jinan, China; ^2^ Laboratory of Translational Gastroenterology, Qilu Hospital, Cheeloo College of Medicine, Shandong University, Jinan, China; ^3^ Advanced Medical Research Institute, Shandong University, Jinan, China; ^4^ Robot Engineering Laboratory for Precise Diagnosis and Therapy of GI Tumor, Qilu Hospital, Cheeloo College of Medicine, Shandong University, Jinan, China; ^5^ Key Laboratory for Experimental Teratology of Ministry of Education, Shandong University, Jinan, China

**Keywords:** inflammatory bowel disease, ulcerative colitis, melatonin, ILC2, microbiota

## Abstract

Levels of type 2 cytokines are elevated in the blood and intestinal tissues of ulcerative colitis (UC) patients in the active phase; this phenomenon indicates the participation of type 2 immune response in UC progression. The beneficial effects of melatonin in dextran sodium sulfate (DSS) and 2,4,6-trinitrobenzene sulfonic acid (TNBS) colitis models have been illustrated, but its role in the oxazolone (Oxa)-induced colitis model (driven by type 2 immune response) remains relatively unknown. We investigated the relationship between melatonin concentration and the severity of UC, revealing a significantly negative correlation. Subsequently, we investigated the effects of melatonin in Oxa-induced colitis mice and the potential underlying mechanisms. Administration of melatonin significantly counteracted body weight loss, colon shortening, and neutrophil infiltration in Oxa-induced colitis mice. Melatonin treatment mitigated Oxa-induced colitis by suppressing type 2 immune response. In addition, melatonin attenuated intestinal permeability by enhancing the expression of ZO-1 and occludin in colitis mice. Interestingly, the protective effect of melatonin was abolished when the mice were co-housed, indicating that the regulation of gut microbiota by melatonin was critical in alleviating Oxa-induced colitis. Subsequently, 16S rRNA sequencing was performed to explore the microbiota composition. Decreased richness and diversity of intestinal microbiota at the operational taxonomic unit (OTU) level resulted from melatonin treatment. Melatonin also elevated the abundance of *Bifidobacterium*, a well-known probiotic, and reduced proportions of several harmful bacterial genera, such as *Desulfovibrio*, Peptococcaceae, and Lachnospiraceae. Fecal microbiota transplantation (FMT) was used to explore the role of microbiota in the function of melatonin in Oxa-induced colitis. Microbiota transplantation from melatonin-treated mice alleviated Oxa-induced colitis, suggesting that the microbiome participates in the relief of Oxa-induced colitis by melatonin. Our findings demonstrate that melatonin ameliorates Oxa-induced colitis in a microbiota-dependent manner, suggesting the therapeutic potential of melatonin in treating type 2 immunity-associated UC.

## Introduction

Inflammatory bowel disease (IBD) is a chronic disease of the gastrointestinal (GI) tract that leads to frequent diarrhea, abdominal pain, weight loss, and impaired GI motility (1). IBD is classified into ulcerative colitis (UC) and Crohn’s disease (CD) based on clinical symptoms (2, 3). The former is the predominant pathological subtype of IBD (4). However, the critical factors for the initiation and development of UC remain elusive (5, 6). Multiple risk factors might contribute to the UC progression, including mutations in some genes such as *Nod2* and *Card9*, abnormal activation of immune cells, and disorders of gut microflora (7–10). Concentrations of type 2 cytokines, such as IL-5 and IL-13, increase in the blood and intestinal tissues of UC patients in the active phase, indicating that type 2 immune response may contribute to UC progression (11, 12). In contrast, secretion of amphiregulin (Areg) by group 2 innate lymphoid cells (ILC2s) is indispensable for tissue repair in colitis, suggesting the complicated roles of type 2 immunity in different phases of UC (13). Oxazolone (Oxa) induces colitis in mice (involving type 2 cytokines), which recapitulates a similar immune response in the active stage of UC patients (14, 15).

Melatonin (*N*-acetyl-5-methoxytryptamine) is mainly secreted by the pineal gland and participates in several physiological processes, such as circadian rhythm management and immune modulation (16–18). In addition to the brain, the GI tract contains melatonin, whose concentration is higher in the GI tract than that in the circulation by 10 to 400 times (19). The secretion of melatonin in the GI tract is controlled by dietary rather than circadian rhythms (20–22). Melatonin in the GI tract is principally produced by enterochromaffin cells (EC), certain types of immune cells, and intestinal commensals (19, 23–25). The liver degrades virtually all of melatonin, suggesting that GI melatonin is confined to the gut–liver axis, instead of circulating into other organs, and that it functions locally (26).

There are a variety of therapeutic options for UC, including immunosuppressive hormones such as glucocorticoids or their analogs, and monoclonal antibodies targeting inflammatory molecules (4). However, adverse effects and low responsiveness limit the utility of these strategies. Therefore, safe and effective treatment of UC requires substantial further investigation. Melatonin affects the permeability, motility, and barrier function of the intestine as an anti-inflammatory agent and antioxidant (27–29). Melatonin ameliorates the inflammatory reaction and colonic injury in experimental colitis induced by both dextran sodium sulfate (DSS) and 2,4,6-trinitrobenzene sulfonic acid (TNBS) in mice and rats (28, 30–32). Clinically, melatonin treatment relieves abdominal pain and diarrhea (27). Notably, melatonin can also change the composition of the gut microbiota (33). In addition, melatonin increases the abundance of *Firmicutes* and *Bacteroides* and reduces the proportion of harmful bacteria in mice (34, 35). However, it remains to be explored whether melatonin affects the microbiota to influence the pathological consequences in UC.

In this study, we demonstrated a negative correlation between the melatonin concentration in colonic tissues and UC progression. We also determined that the levels of key enzymes for melatonin synthesis were diminished in UC patients. Melatonin relieved Oxa-induced colitis in mice, concomitantly suppressing ILC2s in a microbiota-dependent manner. Our findings provide new evidence for the beneficial role of melatonin in type 2 immunity-related colitis.

## Materials and Methods

### Study Patients

This study was approved by the Clinical Ethical Committee of Shandong University (ECSBMSSDU2020-1-035). Each participant signed a written informed consent form prior to participation. The basic patient information is shown in **Table 1**. Eleven patients with UC who underwent colonoscopy for disease surveillance were enrolled. The diagnosis was based on well-established clinical, endoscopic, and histopathological criteria. The healthy controls (HCs) included 17 healthy volunteers who underwent colonoscopy for physical examination. During the colonoscopy, inflamed intestinal mucosal biopsies of UC patients were obtained, as well as normal areas in HCs in the descending colon or sigmoid colon. Collected biopsies were placed in 4% formalin solution or stored at –80°C.

**Table 1:** Demographic of patients with UCs and HCs.

	HC	UC	*p*-Value
Number of subjects	17	11	
Age (years)	49.53 ± 9.44	52.18 ± 15.28	*0.5737*
Gender (male:female)	12:5	8:3	
Weight (kg)	59.76 ± 7.73	58.73 ± 6.77	*0.7193*
BMI (kg/m^2^)	20.75 ± 1.83	20.09 ± 1.63	*0.3463*

HC, healthy controls; UC, ulcerative colitis; BMI, body mass index. p > 0.05, no significant difference.

### Quantification of Melatonin in the Human Colon

Total proteins from colonic biopsies were extracted and centrifuged at 12,000 rpm for 15 min at room temperature. Supernatants were collected, and melatonin concentration was assessed using a human melatonin ELISA kit (CUSABIO, CSB-E08132h) per the manufacturer’s instructions.

### Fixation and Histology

The tissue was then fixed for 24 h at room temperature. Subsequently, a standard dehydration procedure was performed, followed by paraffin embedding. Intestinal tissue was sectioned into 4-µm thin sections for staining. The slides were stained with H&E and scored in a blinded manner, according to a previously published scoring system (36). Briefly, each section was scored based on the following criteria: inflammation, dysplasia, hyperplasia, edema, crypt degeneration, and epithelial damage. The total lesion scores reflect the sum of individual scores.

### RNA Extraction, Reverse Transcription, and qPCR

Total RNA from colonic biopsies was isolated using RNA isolation reagent (Vazyme, R401-01), and the concentration was measured. Total RNA (1 μg) was reverse transcribed into cDNA using the HiScript III RT SuperMix for qPCR (+gDNA wiper) kit (Vazyme, R323-01). Subsequently, cDNA was amplified using the StepOnePlus™ Real-Time PCR System (Thermo Fisher; 4376600). The PCR mixture containing 10 μl of 2× AceQ^®^Universal SYBR qPCR Master Mix (Vazyme, Q511-02), 1 μl of cDNA, forward and reverse primers (0.8 μl each), and diethyl pyrocarbonate (DEPC) water (7.4 μl) was prepared. The primers used for qPCR are listed in **Table 2**. GAPDH was used as the internal standard to normalize target gene expression. For analysis, the 2^−ΔΔCt^ method was used to calculate the mRNA levels of the target genes.

**Table 2:** Primer sequence for qPCR.

	Forward	Reverse
*AANAT*	AGCCACGGTCTCCATACACTCAG	TTTCCTCCTTCCTACACCTCAACAATG
*HIOMT*	GTGGTGGCATTCTGGTAATTGAAAGC	AGAAGAGAGGAGCATGTGGTAGTGG
*GAPDH*	GTCTCCTCTGACTTCAACAGCG	ACCACCCTGTTGCTGTAGCCAA
*II-5*	GATGAGGCTTCCTGTCCCTACT	TGACAGGTTTTGGAATAGCATTTCC
*II-13*	AACGGCAGCATGGTATGGAGTG	TGGGTCCTGTAGATGGCATTGC
*ll-1b*	GAAATGCCACCTTTTGACAGTG	TGGATGCTCTCATCAGGACAG
*Tnfa*	CAGGCGGTGCCTATGTCTC	CGATCACCCCGAAGTTCAGTAG
*Gapdh*	CATGGCCTCCAAGGAGTAAG	CCTAGGCCCCTCCTGTTATT

### Mouse Colitis Model

All animal experiments were carried out in compliance with and approved by the Shandong University Specific Pathogen Free (SPF) animal center. All experimental animal procedures were approved by the Animal Care and Animal Experiments Committee of Shandong University (ECSBMSSDU2020-2-057). Animal studies were conducted in a gender- and age-matched manner. Male C57BL/6 mice aged 6–8 weeks were obtained from Nanjing GemPharmatech Animal Center and maintained in SPF facilities at Shandong University. For the induction of Oxa-induced colitis, mice were anesthetized with 3.5 μl/g of 2% sodium pentobarbital. Oxa (4-ethoxymethylene-2-phenyl-2-oxazolin-5-one) (Sigma Chemical Co., St. Louis, MO) was dissolved in a 1:1 H_2_O/ethanol mixture (50% ethanol). Then, 4.5 μl/g of 3% Oxa solution was administered *via* a 3.5-F catheter. The catheter was inserted 4 cm proximal to the anal verge, and the Oxa was injected. To ensure the distribution of Oxa within the entire colon, mice were held in a vertical position for 5 min after the injection. For melatonin treatment, mice were administered 50 mg/kg of melatonin (experimental group) or solvent (control group) daily (via gavage) for 1 week before induction of colitis according to published articles (37–39). Melatonin was purchased from MedChemExpress (CAS Number: 73-31-4) and dissolved in dimethyl sulfoxide (DMSO).

### Residential Model of Mice

Separated mice were obtained from Nanjing GemPharmatech Animal Center, fed in separate cages, and handled separately. Co-housed mice were kept together in one cage for at least 7 days before melatonin treatment.

### Antibiotic Treatment and Fecal Microbiota Transplantation

All the mice used in the fecal microbiota transplantation (FMT) study came from the same batch and were kept together at least 7 days before the experiment. During FMT, the housing environment, the same as before, was strictly controlled to ensure consistency. Mice were administered daily (via gavage) with 200 μl of autoclaved water supplemented with antibiotics (ampicillin 1 g/L, gentamicin 1 g/L, metronidazole 1 g/L, neomycin 1 g/L, and vancomycin 0.5 g/L). After 1 week, antibiotic treatment was stopped, and the mice were recolonized by FMT, receiving stools from melatonin-treated mice and controls. Mice received stools daily for 1 week by oral gavage before Oxa colitis was induced.

### Western Blotting

Total proteins of colon tissue from Oxa-colitis mice were extracted. Proteins were separated by 10% sodium dodecyl sulfate–polyacrylamide gel electrophoresis and transferred onto a polyvinylidene fluoride membrane. The membrane was incubated with the primary antibody at 4°C overnight and then incubated with the secondary antibody for 60 min at room temperature. Immunoblots were detected using an enhanced chemiluminescent substrate (Millipore). Antibodies: anti-ZO-1 antibody (1:1,000; Thermo Fisher), anti-occludin antibody (1:1,000; Thermo Fisher), anti-GAPDH antibody (1:5000; ProteinTech, USA), anti-claudin-1 antibody (1:1,000; Thermo Fisher), anti-claudin-5 antibody (1:1,000; Abcam), and goat anti-rabbit IgG (1:5,000; Zhongshan Gold Bridge, Beijing, China).

### Metagenomic Analysis of Microbiome

A pellet of mouse feces was collected after melatonin treatment and stored at –80°C until further use. 16S rRNA sequencing was performed by Shanghai Majorbio, and the data were analyzed on the Majorbio cloud platform. DNA was isolated using the E.Z.N.A.^®^ soil kit (Omega Bio-Tek, Norcross, GA, USA), and V3–V4 was amplified by PCR. The primers were 338F (5′-ACTCCTACGGGAGGCAGCAG-3′) and 806R (5′-GGACTACHVGGGTWTCTAAT-3′). PCR products were then sequenced using the Illumina MiSeq platform (Illumina, San Diego, USA) and analyzed using UPARSE software (version 7.1).

### Isolation of Lamina Propria Lymphocytes (LPLs) and Flow Cytometry

Isolation of intestinal lamina propria cells and flow cytometry were conducted as previously described (40). To isolate lymphocytes from the lamina propria, the intestinal segment (approximately 0.5 cm) was digested in a complete Roswell Park Memorial Institute (RPMI) medium containing DNase I (150 μg/ml, Sigma) and collagenase VIII (300 U/ml, Sigma) at 37°C in a 5% CO_2_ incubator for 1.5 h. The digested segments were ground and filtered through a 100-μm cell strainer. The cells were collected from the interphase of an 80% and 40% Percoll gradient after spinning at 2,500 rpm for 15 min at room temperature. The CD16/32 antibody (eBioscience) was used to block the Fc receptors before surface staining. Lymphocytes isolated from the intestinal lamina propria were stained with antibodies against the following markers: GATA3 (PB), RORγt (PE), IL-13 (Alexa Fluor 488), IL-5 (APC), IL-22 (APC), CD45.2 (AF700), CD127 (PE), KLRG1 (PerCP-Cy5.5), CD11b (APC-Cy7), and Ly6G (FITC). Lin comprised APC-Cy7 or APC-eFluor 780-CD3, CD5, CD19, B220, Ly6G, FcϵR1, CD11c, CD11b, Ter119, NK1.1, and CD16/CD32. For cytokine staining, cells were stimulated with 50 ng/ml of phorbol myristate acetate (PMA) and 500 ng/ml of ionomycin for 2 h, and Brefeldin A (2 µg/ml) was added 2 h before cells were harvested. Live and dead cells were identified using the Live and Dead Violet Viability Kit or Zombie Aqua Fixable Viability Kit (BioLegend).

### Sorting, Culture, and Treatment of ILC2s *In Vitro*

ILC2s (Lin^−^KLRG1^+^CD127^+^) were sorted by flow cytometry (Beckman, Moflo Astrios EQ). Sorted ILC2s were cultured in IMDM medium containing 10% fetal bovine serum (FBS), 10 ng/ml of IL-2, 10 ng/ml of IL-7, 10 ng/ml of IL-25, and 10 ng/ml of IL-33. Purified ILC2s were treated with melatonin for 4 or 16 h, and cytokine production by ILC2s was assessed by flow cytometry.

### Statistical Analysis

Unless otherwise noted, statistical analyses were performed using the unpaired two-tailed Student’s *t*-test with GraphPad Prism. Results are expressed as mean ± SD (*p < 0.05, **p < 0.01, ***p < 0.001, ****p < 0.0001).

## Results

### Melatonin in the Colon Reduces and Negatively Correlates With Disease Severity in Ulcerative Colitis Patients

**Figure 1:**
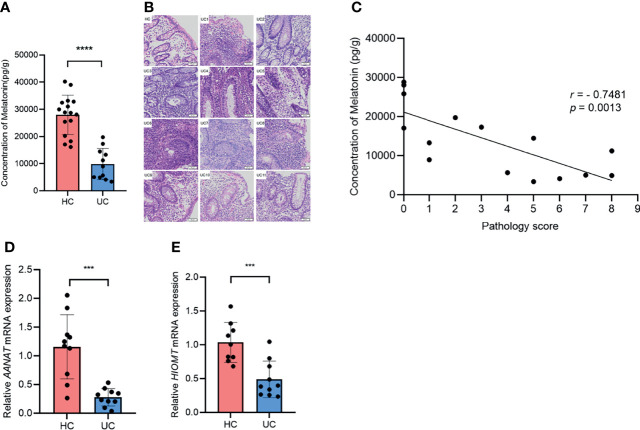
Melatonin in the colon decreases and negatively associates with disease severity in ulcerative colitis (UC) patients. **(A)** Melatonin concentrations in healthy control (HC) and UC patients were detected by ELISA. **(B)** Representative H&E staining of colonic sections (×20). **(C)** Correlation of melatonin concentration and histological severity scores of all 11 UC patients and 4 random HCs. **(D, E)** *AANAT* and *HIOMT* mRNA expression levels in HCs and UC patients (all values were normalized to the HC group). ***p < 0.001; ****p < 0.0001.

To investigate the role of melatonin in UC, we measured the concentration of melatonin in the descending and sigmoid colon of UC patients and HCs. Compared with those in HCs, the levels of melatonin decreased drastically in UC patients (**Figure 1A**). H&E staining indicated that the pathological consequence of UC was more severe than that of HCs (**Figure 1B**). Notably, a negative association emerged between melatonin concentration and histologic severity in all 11 patients with UC and 4 random HCs (**Figure 1C**). In addition, the mRNA levels of *AANAT* and *HIOMT*, the key enzymes in the melatonin biosynthesis pathway, were significantly less in patients with UC than in HCs (**Figures 1D, E**). Taken together, these data suggest that the level of melatonin decreases in UC patients and correlates negatively with disease severity, indicating that melatonin may be involved in UC progression.

### Melatonin Mitigates Oxazolone-Induced Colitis

**Figure 2:**
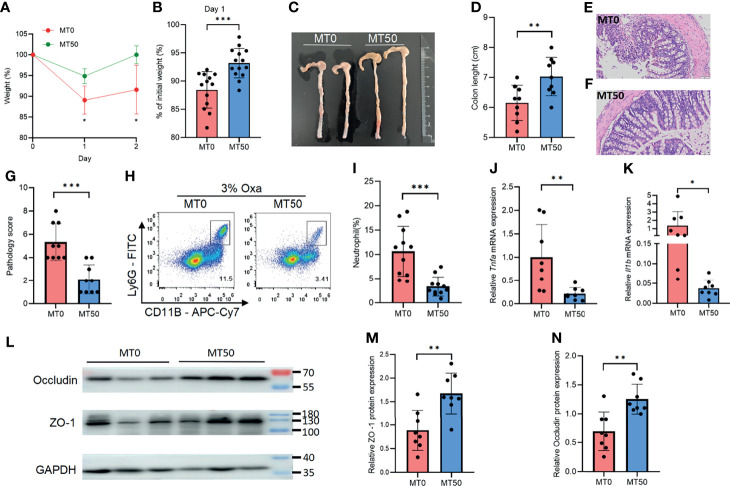
Melatonin mitigates oxazolone (Oxa)-induced colitis. **(A)** Body weight changes after 3% Oxa-treated (day 0). **(B)** Weight changes at day 1. **(C, D)** Colon length at day 1. **(E–G)** Representative H&E staining (×20) and pathological score of the inflamed gut epithelium. **(H, I)** Flow cytometry analysis of colonic CD45^+^CD11b^+^Ly6G^+^ neutrophil subsets in melatonin-treated and melatonin-untreated mice at day 1 after 3% oxazolone enema. **(J, K)** qPCR of *Tnfa* and *Il-1b* mRNA in colonic tissues in melatonin-treated and melatonin-untreated mice at day 1 after 3% oxazolone enema (all values were normalized to the melatonin-untreated group). **(L)** Immunoblot for ZO-1 and occludin in colonic tissue and **(M, N)** the densitometric analysis of ZO-1 and occludin immunoblot of melatonin-treated and melatonin-untreated mice. Data are representative of at least two independent experiments. Data are shown as mean ± SD. *p < 0.05; **p < 0.01; ***p < 0.001.

Oxa-induced colitis, an experimental animal colitis model, is mediated by the type 2 immune response. The experimental model shows pathological similarity to UC in humans, particularly regarding the elevated levels of type 2 cytokines. To explore the effect of melatonin on colitis *in vivo*, Oxa was administered to mice by enema after anesthesia. Compared with HC, Oxa-induced colitis mice showed significant inflammation after enema (**Supplementary Figures 1A–D**). Melatonin treatment at a daily dose of 50 mg/kg for 7 days (MT50) markedly ameliorated Oxa-induced colitis in mice, in terms of less body weight loss and longer colon length (**Figures 2A–D**). H&E staining revealed that melatonin treatment prevented intestinal inflammation, and the pathology score of the colon in the MT50 group was lower than that in the MT0 group (**Figures 2E–G**). Next, we examined inflammatory cell infiltration by flow cytometry and the production of inflammatory cytokines by qPCR. Melatonin treatment resulted in a significant reduction of CD11b^+^Ly6G^+^ neutrophils compared with the control group (MT0), and a decreased production of inflammatory cytokines, such as *Tnfa* and *Il-1β* (**Figures 2H–K**). Accordingly, lower expression levels of tight junction-associated proteins (e.g., occludin and ZO-1) correlate with higher levels of inflammation and reflect worsened disease; these proteins were significantly upregulated in the MT50 group (**Figures 2L–N**). These results indicate that melatonin alleviates Oxa-induced colitis, as indicated by a decreased inflammatory response and elevated intestinal integrity.

### Melatonin Inhibits Type 2 Immunity in Oxazolone-Induced Colitis

**Figure 3:**
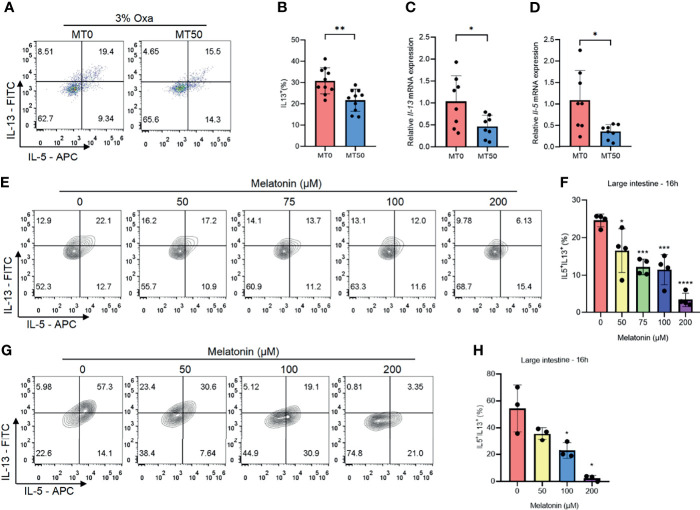
Melatonin inhibits type 2 immunity in oxazolone (Oxa)-induced colitis. **(A, B)** Flow cytometry analysis of IL-5 and IL-13 in colonic CD45^+^Lin^−^GATA3^+^ ILC2s and quantification of IL-13 in colonic ILC2s in melatonin-treated and melatonin-untreated mice at day 1 after 3% oxazolone enema. **(C, D)** qPCR of *Il-5* and *Il-13* mRNA of colonic tissue in melatonin-treated and melatonin-untreated mice at day 1 after 3% oxazolone enema (all values were normalized to the melatonin-untreated group). **(E, F)** Representative flow cytometry plots and quantification of large intestinal CD45^+^Lin^−^GATA3^+^ ILC2 function treated with melatonin for 16 h in LPLs *in vitro*. **(G, H)** Representative flow cytometry plots and quantification of purified large intestinal CD45^+^Lin^−^CD127^+^KLRG1^+^ ILC2 function treated with melatonin for 16 h *in vitro*. Data are representative of at least two independent experiments. Data are shown as mean ± SD. *p < 0.05; **p < 0.01; ***p < 0.001; ****p < 0.0001.

Owing to Oxa-induced colitis being mediated by type 2 immunity, the effect of melatonin on the secretion of type 2 inflammatory cytokines was assessed, and melatonin treatment markedly suppressed the production of IL-5 and IL-13 in the colon of the MT50 group (**Figures 3A, B** and **Supplementary Figure 2A**). Additionally, the mRNA expression of IL-5 and IL-13 was also inhibited by melatonin (**Figures 3C, D**). Notably, IL-5 and IL-13 were mainly secreted by CD45^+^Lin^−^GATA3^+^ cells in the colon of Oxa-induced colitis (**Supplementary Figure 2B**), suggesting that ILC2s were the main producers of type 2 cytokines rather than Th2 cells. Melatonin significantly decreased the production of IL-5 and IL-13 in a dose- and time-dependent manner in both large and small intestinal ILC2s isolated from the lamina propria *in vitro* (**Figures 3E, F** and **Supplementary Figures 2C–F**). In contrast, ILC3s, another subset of ILC2s, were unaffected by melatonin (**Supplementary Figures 2G, H**). To eliminate the possibility that other compartments of LPLs mediate the effect of melatonin, the sorted ILC2s from the mouse large intestine were treated with melatonin, showing that the secretion of IL-5 and IL-13 by ILC2s was significantly suppressed by melatonin (**Figures 3G, H**). Collectively, these results suggest that melatonin directly inhibits ILC2s, which might be responsible for its role in improving Oxa-induced colitis.

### Effect of Melatonin on Oxazolone-Induced Colitis Depends on Gut Microbiota

**Figure 4:**
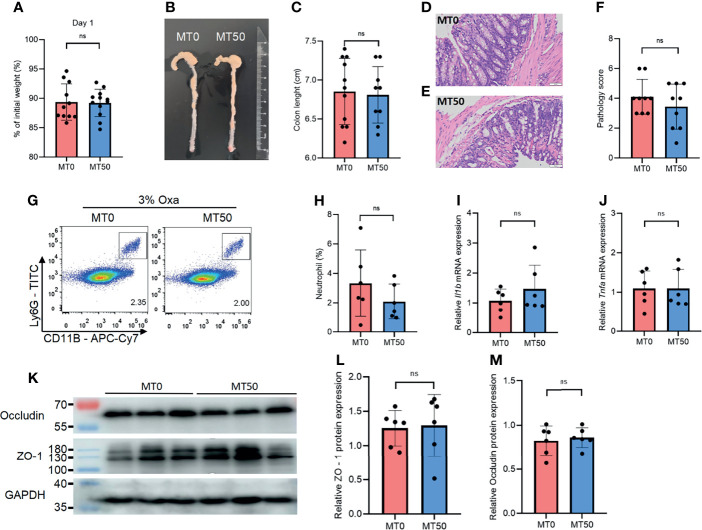
Effect of melatonin on oxazolone (Oxa)-induced colitis depends on gut microbiota. **(A)** Weight changes and colon length **(B, C)** in co-housed mice at day 1 after 3% oxazolone enema. **(D–F)** Representative H&E staining of colonic sections (×20) and pathological score of inflamed colonic tissue. **(G, H)** Flow cytometry analysis of colonic CD45^+^CD11b^+^Ly6G^+^ neutrophil subsets in co-housed mice at day 1 after 3% oxazolone enema. **(I, J)** qPCR of *Tnfa* and *Il-1b* mRNA in colonic tissues in co-housed mice at day 1 after 3% oxazolone enema (all values were normalized to the melatonin-untreated group). **(K–M)** Immunoblot and densitometric analysis of ZO-1 and occludin in co-housed mice. Data are representative of at least two independent experiments. Data are shown as mean ± SD. ns, no significance.

Accumulating evidence indicates that there is a link between gut microbiota and intestinal inflammation in UC, in line with the crucial function of microbial colonization of the immune system. We noticed that the protective effect of melatonin on weight loss and colonic shortening in Oxa-induced colitis was abolished in co-housed mice, suggesting that the microbiome is the key target of melatonin in controlling gut inflammation (**Figures 4A–C**). There was no significant difference in the histological severity of colitis and neutrophil infiltration between the MT0 and MT50 groups from co-housed mice (**Figures 4D–H**). Accordingly, microbiota communication also abolished the effect of melatonin on the expression of inflammatory cytokines and tight junction-associated proteins (**Figures 4I–M**). Together, the regulation of gut microbiota by melatonin is critical for alleviating Oxa-induced colitis.

### Melatonin Alters Microbiota Composition

**Figure 5:**
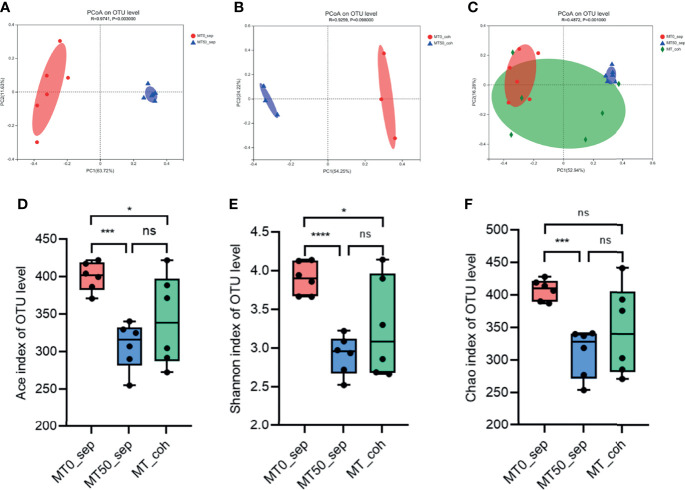
Melatonin alters microbiota composition in alpha and beta diversity. **(A)** Principal coordinate analysis (PCoA) of fecal microbiota in separately housed mice treated or untreated with melatonin for 7 days. **(B)** PCoA of fecal microbiota in co-housed mice treated or untreated with melatonin for 7 days. **(C)** PCoA of fecal microbiota among separately housed melatonin-treated mice, separately housed melatonin-untreated mice, and co-housed mice. PCoA is based on binary Jaccard distance. ANOSIM and 999 times permutation tests were utilized. **(D–F)** Shannon, Chao, and Ace indices of operational taxonomic unit (OTU) levels. Data are expressed as mean ± SD; statistical significance was determined by a two-sided Student’s t-test or one-way ANOVA. *p < 0.05; ***p < 0.001; ****p < 0.0001. ns, no significance.

**Figure 6:**
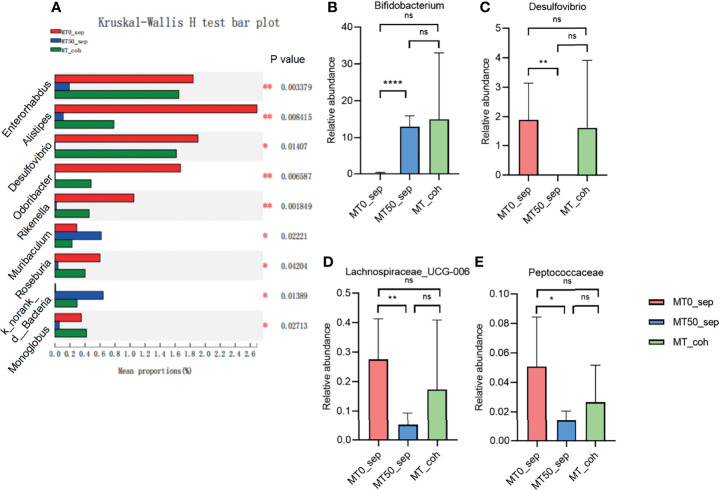
Melatonin regulates certain microbiota on genus level. **(A)** Kruskal–Wallis H test for differences between separated and co-housed mice for 7-day melatonin treatment on genus levels. **(B–E)** Relative abundance of *Bifidobacterium*, *Desulfovibrio*, Lachnospiraceae_UCG-006, and Peptococcaceae on genus levels. Data are expressed as mean ± SD. *p < 0.05; **p < 0.01; ****p < 0.0001. ns, no significance.

Given that the effect of melatonin on colitis induced by Oxa is mediated by gut microbiota, we collected feces of separately housed or co-housed mice treated or untreated with melatonin, and 16S rRNA gene high-throughput pyrosequencing was performed. Principal coordinate analysis (PCoA) was used to assess the overall microbiome composition (beta diversity), which showed significant differences in clustering between vehicle- and melatonin-treated groups of separately housed mice, while there was no significant difference between co-housed mice treated or untreated with melatonin (**Figures 5A–C**). The richness and diversity of flora in melatonin-treated mice from separately housed mice were significantly lower than those in the melatonin-treated group according to the Ace, Chao, and Shannon indices of operational taxonomic unit (OTU) level (**Figures 5D–F**). There was no significant difference in the richness and diversity between melatonin-untreated separately housed mice and co-housed mice. The abundance of Verrucomicrobiota and Actinobacteria in the melatonin-treated separately housed group was greater than that in the other groups at the phylum level (**Supplementary Figure 3A**). At the genus level, the melatonin-treated group, compared with the melatonin-untreated group, showed significant suppression of proportions of *Desulfovibrio*, Lachnospiraceae, and Peptococcaceae and increased abundance of *Bifidobacterium* (**Figures 6A–E** and **Supplementary Figure 3B**). Additionally, the significant difference of the main genus between the melatonin-treated and melatonin-untreated groups is shown as a circos plot (**Supplementary Figure 3C**). Taken together, melatonin treatment causes significant changes in the intestinal microbiota, some of which are erased by co-housing.

### Microbiota Transplantation From Melatonin-Treated Mice Alleviates Oxazolone-Induced Colitis

**Figure 7:**
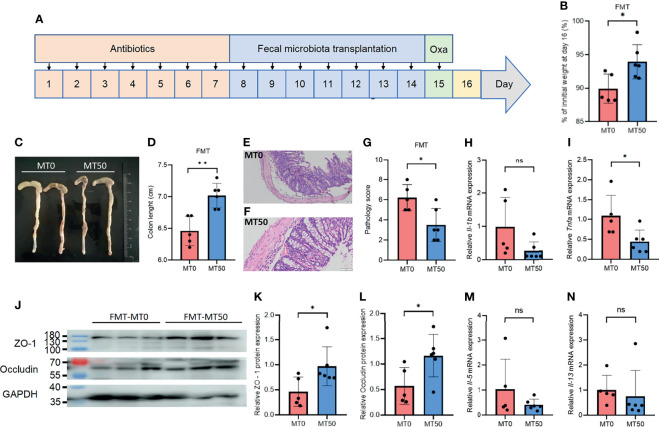
Microbiota transplantation from melatonin-treated mice alleviates oxazolone (Oxa)-induced colitis. **(A)** The process of fecal microbiota transplantation. The mice were treated with antibiotics for 7 days to clear the gut microbiota (day 1 to day 7) and transplanted feces from 7-day melatonin-treated or melatonin-untreated mice (day 8 to day 14) before 3% oxazolone enema (day 15). Mice were sacrificed on day 16. **(B)** Body weight changes in fecal microbiota transplantation (FMT) mice at day 16. **(C, D)** Colon length in FMT mice at day 16. **(E–G)** Representative H&E staining (×20) and pathological score of the inflamed gut epithelium. **(H, I)** qPCR of *Tnfa* and *Il-1b* mRNA in colonic tissues in FMT mice at day 16 after 3% oxazolone enema (all values were normalized to MT0 group). **(J–L)** Immunoblot and densitometric analyses of ZO-1 and occludin in FMT mice at day 16 after 3% oxazolone enema. **(M, N)** qPCR of *Il-5* and *Il-13* mRNA in colonic tissues in FMT mice at day 16 after 3% oxazolone enema (all values were normalized to MT0 group). Data are representative of two independent experiments. Data are shown as mean ± SD. *p < 0.05; **p < 0.01; ***p < 0.001. ns, no significance.

As melatonin significantly changed the composition of the bacterial community, we sought to explore whether FMT from melatonin-treated mice could alleviate Oxa-induced colitis. Fecal samples collected from melatonin-treated or untreated mice were transplanted into antibiotic-treated mice, and Oxa was administered to the mice with fecal transplantation to induce colitis (**Figure 7A**). Antibiotic-treated mice receiving melatonin-treated feces showed marked relief of Oxa-induced colitis, including weight loss, colonic shortening, and histological severity (**Figures 7B–G**), which coincided with a decrease in the level of the inflammatory cytokine TNFα, but not in the level of IL-1β, in the colon (**Figures 7H, I**). Accordingly, the expression of tight junction-associated proteins, occludin and ZO-1, was markedly upregulated in mice transplanted with microbiota from melatonin-treated mice (**Figures 7J–L**), while there was no significant difference of type 2 inflammatory cytokines between the two groups (**Figures 7M, N**). These results indicate that melatonin alleviates Oxa-induced colitis by regulating microbiota.

## Discussion

UC is a common chronic GI disease with histopathological features of tissue damage, lymphocyte infiltration, epithelial destruction, and ulcer formation (41). UC is associated with recurrent episodes and is often associated with mental disorders such as anxiety and depression (42). Melatonin improves emotional states in patients; thus, it is often used as an auxiliary medication in the treatment of many diseases. No severe adverse effects are observed (18, 25, 43). The concentration of melatonin in venous blood is significantly reduced in patients with UC (44). Notably, urinary excretion of 6-sulfatoxymelatonin, a metabolite of melatonin in the liver, is greater in UC patients than in HC, suggesting an increase in intestinal melatonin level (44, 45). In this study, we used biopsy specimens from human descending or sigmoid colon to assess the concentration of melatonin by ELISA, which clarified the fact that melatonin levels in UC patients gradually decreased with the severity of inflammation, revealing a regulatory potential of melatonin during UC.

Melatonin is highly enriched in the digestive tract, especially in the intestine, where its concentration is greater than that in the circularity system by a factor of 10–400 (19). A major amount of intestinal melatonin is reported to be secreted by EC, which are neuroendocrine-immune cells that are critical for intestinal homeostasis (31, 46). EC is capable of sensing bacterial metabolites or neurotransmitters in the intestine and responds accordingly. Due to the drastic alteration of the intestinal microenvironment during UC, EC showed a significant reduction in sensory function, but enhanced secretion, such as 5-HT and antimicrobial peptides (47). Since EC is the major producer of melatonin in the gut, decreased melatonin levels in UC patients highlight the role of EC in the context of colitis, indicating that comprehensive and in-depth research on the function of EC remains to be investigated.

There are three main experimental animal models of colitis: the DSS, TNBS, and Oxa models (30, 48, 49). Melatonin relieves DSS colitis through epithelial components, such as goblet cells or stem cells, and affects the intestinal microbiota to improve oxidative stress in DSS colitis (50, 51). In addition, melatonin attenuates TNBS-induced colitis through a melatonin receptor-independent pathway in mice and relieves TNBS colitis through the TLR4/MyD88/NF-κB signaling pathway in rats (30). Nevertheless, the effect of melatonin on type 2 immunity-dependent colitis needs to be documented, as levels of type 2 cytokines, including IL-5 and IL-13, increase in UC patients (11, 12). Oxa-induced colitis is a type 2 immunity-based disease with increased expression levels of IL-4, IL-5, and IL-13 (14). Our data show that melatonin significantly mitigates Oxa-induced colitis, providing new evidence on the beneficial function of melatonin in the field of experimental colitis. Because of the rapid progression of Oxa-induced colitis and a longer melatonin duration, only pretreatment with melatonin was used *in vivo* during this report, which was a limitation of this study.

Melatonin regulates a variety of immune cells, such as T helper cells, Treg cells, B cells, macrophages, and mast cells, but it remains unclear whether melatonin affects ILCs (25). Our results reveal that ILC2-mediated type 2 immunity functions as a pro-inflammatory cell compartment in Oxa-induced colitis, and melatonin inhibits ILC2s in the disease. Intriguingly, melatonin inhibits ILC2s but not ILC3s, indicating a specific regulation of ILC2s by melatonin. Melatonin acts on target cells *via* three types of receptors located in the cell membrane or nucleus, including the classical membrane receptor MT1/MT2, the nuclear receptor superfamily RZR/RORα, and quinone reductase 2 (Nqo2, also called MT3) (25, 52, 53). The expression of melatonin receptors MT1 and MT2 in ILC2s is relatively small, and RZR/RORα is a positive regulator of ILC2s (13, 54). Therefore, MT3 might be responsible for the regulation of ILC2s by melatonin. The mechanism by which melatonin controls ILC2s requires further investigation. Notably, co-housing of mice abolished the relief of Oxa-induced colitis by melatonin, indicating that the microbiota overwhelms other parts in mediating the beneficial effects of melatonin.

The effects of melatonin on the microbiota have been studied in different contexts. The abundance of *Akkermansia muciniphila* increased and the abundance of *Bacteroides massiliensis* decreased in stressed mice after melatonin administration (55). Oral melatonin supplementation alleviated lipid accumulation and reversed gut microbiota dysbiosis in high-fat diet-fed mice, along with an altered abundance of *Bacteroides* and *Alistipes (* 56). Melatonin controls the gut microbiota of mice *via* Reg3β (57). Notably, different methods (gavage or intraperitoneal injection) and various doses of melatonin lead to different changes in the gut flora. In this study, we observed that melatonin reduced the amount of certain harmful bacterial genera such as *Desulfovibrio*, Peptococcaceae, and Lachnospiraceae and increased the abundance of beneficial genera such as *Bifidobacterium*. In accordance, *Bifidobacterium*, a well-known probiotic, colonizes the intestinal mucus layer and can modulate mucus production by goblet cells. It adheres to intestinal mucus and secretes metabolites that upregulate the major mucin MUC2 to maintain intestinal integrity (58). As an over-the-counter (OTC) medicine, *Bifidobacterium* improves UC clinically and pathologically (59, 60). This study found that melatonin was able to stimulate *Bifidobacterium* in Oxa-induced colitis, which may be an important pathway on its anti-colitic effect. In contrast, *Desulfovibrio* and Peptococcaceae are opportunistic pathogens, which are positively related to gut inflammation (59). The severity of colitis can be mitigated by reducing them (60, 61), which provides us another potential mechanism for the treatment of colitis by melatonin. Additionally, Lachnospiraceae_UCG-006 is related to short-chain fatty acid metabolism, which may participate in the process of colitis and liver inflammation (62, 63). Accordingly, the transplantation of feces from melatonin-treated mice mitigates Oxa-induced colitis, validating that melatonin alleviates type 2 immunity-related colitis in a microbiota-dependent manner.

In conclusion, our study demonstrates that melatonin mitigates type 2 immune response-induced colitis and uncovers the indispensable role of microbiota in this process. Further studies are needed to elucidate how melatonin shapes gut microflora and controls ILC2s mechanistically, as well as how to use melatonin against UC clinically. The identification of melatonin-regulated commensals could provide new therapeutic strategies for the treatment of UC.

## Conclusion

Our findings demonstrate that melatonin ameliorates Oxa-induced colitis in a microbiota-dependent manner, suggesting the therapeutic potential of melatonin in UC by targeting type 2 immune response.

## Data Availability Statement

The datasets presented in this study can be found in online repositories. The names of the repository/repositories and accession number(s) can be found below: https://www.ncbi.nlm.nih.gov/, PRJNA752219.

## Ethics Statement

The studies involving human participants were reviewed and approved by the Clinical Ethical Committee of Shandong University (ECSBMSSDU2020-1-035). The patients/participants provided their written informed consent to participate in this study.

## Author Contributions

Z-xZ, S-yL, and Y-qL designed and performed the experiments, analyzed the data, and wrote the paper. Y-yC wrote the paper. XY, G-jK, and JL performed the experiments and analyzed the results. M-qZ, JS, Y-jZ, R-cZ, and B-yJ contributed to the concept and technical support. B-cF and X-lZ supervised the project. All authors participated in revising the manuscript.

## Funding

This study was supported by the National Key R&D Program of China (2020YFA0804400), the National Natural Science Foundation of China (81873550, 82070552, 81800462, and 82071854), and the Youth Interdiscipline Innovative Research Group of Shandong University (2020QNQT009). This study is also supported by the Taishan Scholars Program of Shandong Province and the National Clinical Research Center for Digestive Diseases supporting technology project (2015BAI13B07).

## Conflict of Interest

The authors declare that the research was conducted in the absence of any commercial or financial relationships that could be construed as a potential conflict of interest.

## Publisher’s Note

All claims expressed in this article are solely those of the authors and do not necessarily represent those of their affiliated organizations, or those of the publisher, the editors and the reviewers. Any product that may be evaluated in this article, or claim that may be made by its manufacturer, is not guaranteed or endorsed by the publisher.

## Supplementary Material

Supplementary Figure 1 (Related to **Figure 2**) Inflammation presents in oxazolone-induced colitis mice compared with healthy mice. **(A, B)** Representative HE staining (10×) and pathological score of healthy and inflamed gut epithelium. **(C, D)** qPCR of *Tnfa* and *Il-1b* mRNA in colonic tissues in healthy controls and Oxa-induced colitis (all values were normalized to healthy controls).

Supplementary Figure 2 (Related toto **Figure 3**) Melatonin inhibits ILC2s but not ILC3s in LPLs *in vitro*. **(A)** Flow cytometry analysis of IL-5 and IL-13 in colonic CD45^+^Lin^—^GATA3^+^ ILC2s (secrete IL-5 and IL-13 mainly) and CD45^+^Lin^—^GATA3^—^ ILC1s & ILC3s (IL-5 and IL-13 are not secreted) in Oxa-induced colitis. That indicated the right gating in our gating strategy. **(B)** Representative flow cytometry analysis of IL-13 secretion at day 1 after 3% Oxa-treated. **(C, D)** Representative flow cytometry plots and quantification of large intestinal and **(E, F)** Small intestinal CD45^+^Lin^—^GATA3^+^ ILC2s function treated by melatonin for 4 hours in LPLs *in vitro*. **(G, H)** Quantification of small and large intestinal CD45^+^Lin^—^RORγt^+^ ILC3s function treated by melatonin for 16 hours in LPLs *in vitro*. Data are representative of at least two independent experiments. Data are shown as mean ± SD. * *p* < 0.05; ** *p* < 0.01; *** *p* < 0.001.

Supplementary Figure 3 (Related to **Figure 5**) Melatonin alters microbiota composition. **(A)** Community bar plot analysis of fecal microbiota from separated and co-housed mice for 7-day-melatonin-treatment on Phylum levels. **(B)** Heatmap of different genus between separated and co-housed mice for 7-day-melatonin-treatment. **(C)** Contribution of fecal microbiota from separated and co-housed mice for 7-day-melatonin-treatment on genus levels in Circos plot.
