# Supplementary figures and images for: Melatonin Mitigates Oxazolone-Induced Colitis in Microbiota-Dependent Manner
Source: Front Immunol. 2022 Jan 18;12:783806. doi: 10.3389/fimmu.2021.783806 (PMC8805729; doi:10.3389/fimmu.2021.783806)

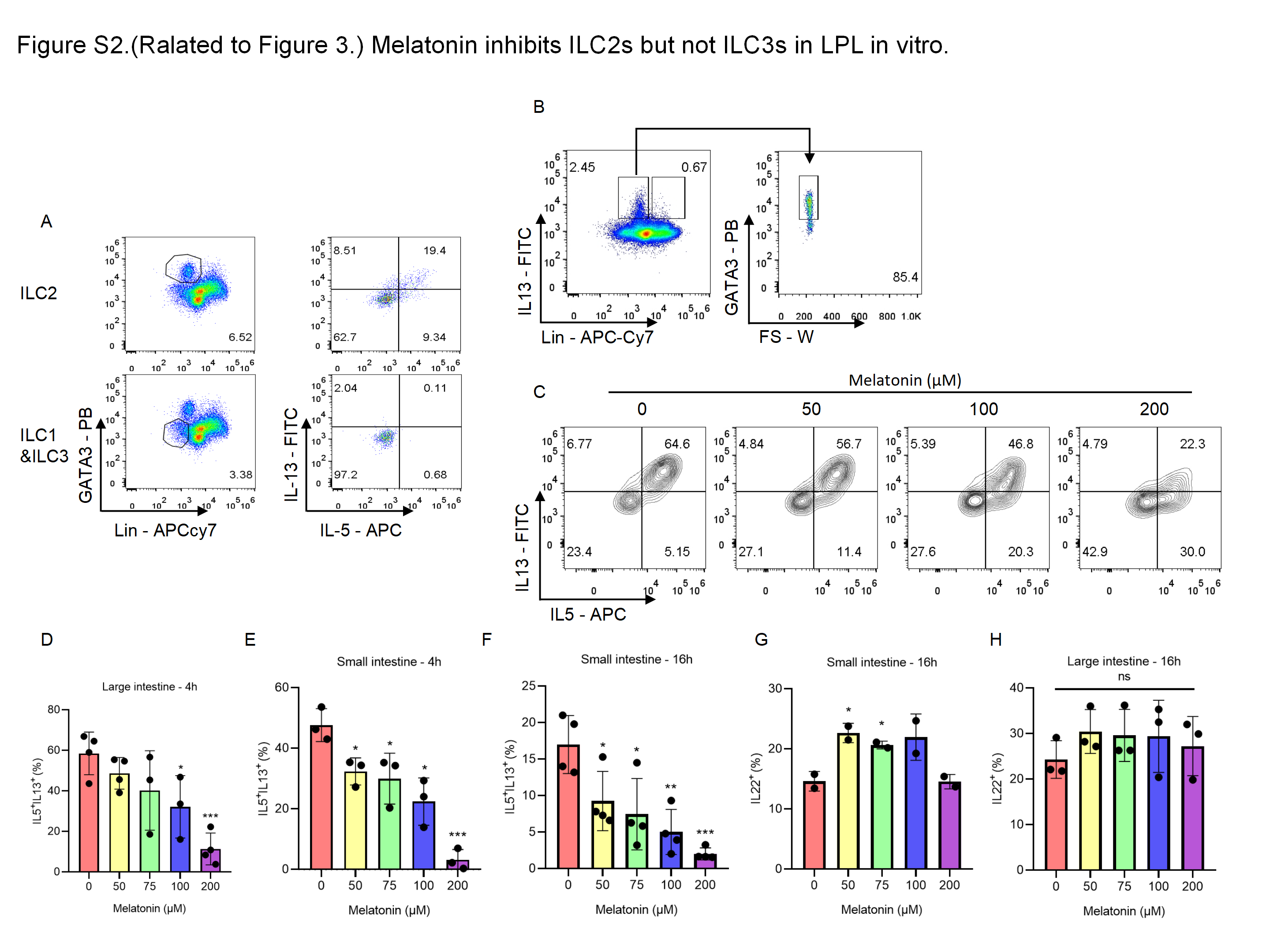

Supplement: Supplementary Figure 1 — (Related to Figure 2 ) Inflammation presents in oxazolone-induced colitis mice compared with healthy mice. (A, B) Representative HE staining (10×) and pathological score of healthy and inflamed gut epithelium. (C, D) qPCR of Tnfa and Il-1b mRNA in colonic tissues in healthy controls and Oxa-induced colitis (all values were normalized to healthy controls). [file DataSheet_1.zip › Supplemental figure 2.tif]

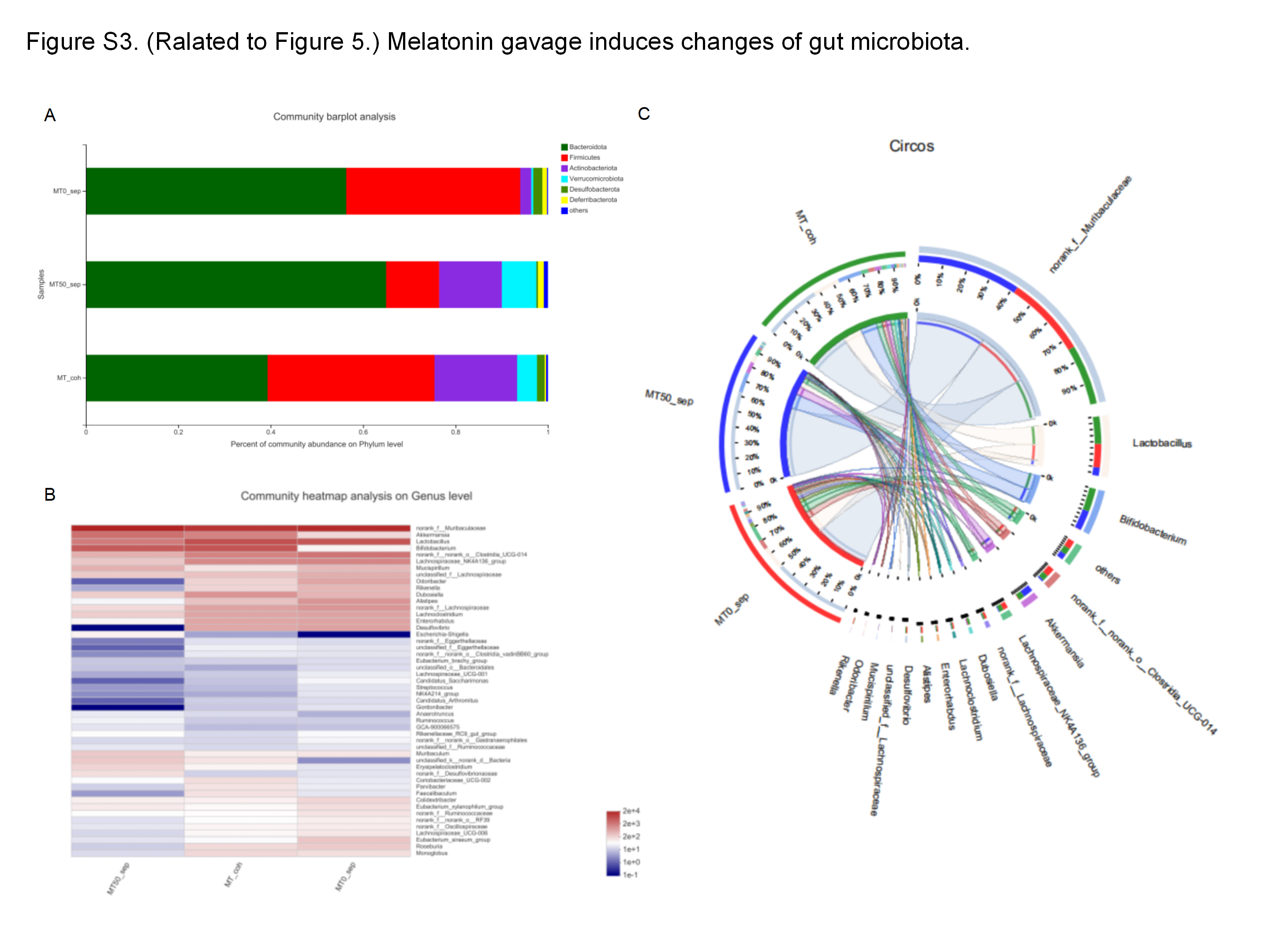

Supplement: Supplementary Figure 1 — (Related to Figure 2 ) Inflammation presents in oxazolone-induced colitis mice compared with healthy mice. (A, B) Representative HE staining (10×) and pathological score of healthy and inflamed gut epithelium. (C, D) qPCR of Tnfa and Il-1b mRNA in colonic tissues in healthy controls and Oxa-induced colitis (all values were normalized to healthy controls). [file DataSheet_1.zip › Supplemental figure 3.tif]

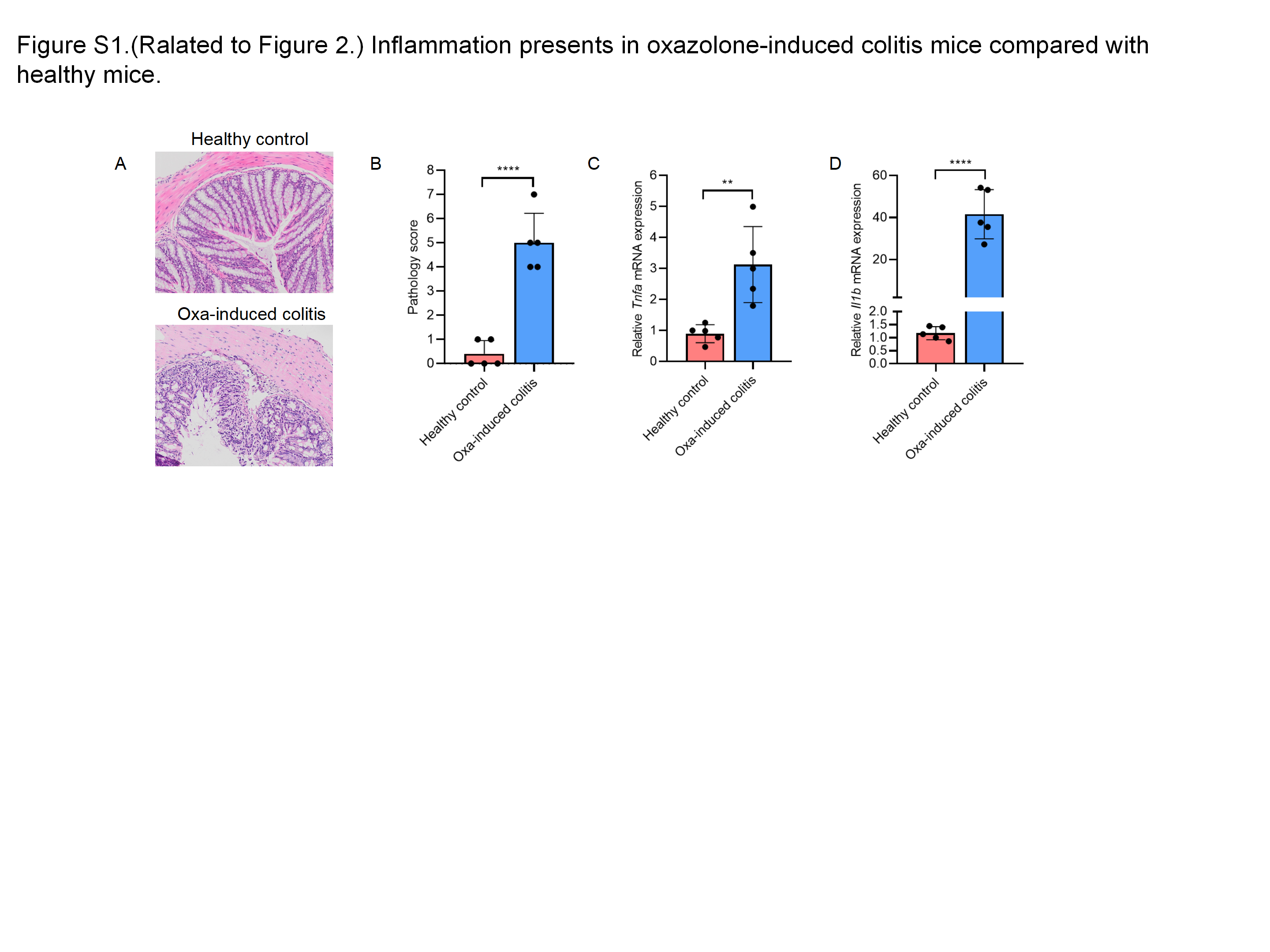

Supplement: Supplementary Figure 1 — (Related to Figure 2 ) Inflammation presents in oxazolone-induced colitis mice compared with healthy mice. (A, B) Representative HE staining (10×) and pathological score of healthy and inflamed gut epithelium. (C, D) qPCR of Tnfa and Il-1b mRNA in colonic tissues in healthy controls and Oxa-induced colitis (all values were normalized to healthy controls). [file DataSheet_1.zip › Supplemental figure 1.tif]
